# Early revision rate in robotic-assisted versus conventional total knee arthroplasty: a prospective cohort study 2021–2023 from the Finnish Arthroplasty Register

**DOI:** 10.2340/17453674.2026.46462

**Published:** 2026-09-03

**Authors:** Kasperi J ALAKYLÄ, Emil SYLVESTERSSON, Mikko S VENÄLÄINEN, Antti P ESKELINEN, Antti JAROMA, Outi VÄYRYNEN, Juha KUKKONEN, Keijo T MÄKELÄ

**Affiliations:** 1 Department of Orthopaedics and Traumatology, Turku University Hospital, and University of Turku, Turku; 2 Department of Medical Physics, Turku University Hospital and University of Turku, Turku; 3 Coxa Hospital for Joint Replacement, Tampere; 4 Department of Orthopaedics and Traumatology, Kuopio University Hospital, Kuopio; 5 Division of Operative Care, Department of Orthopaedic and Trauma Surgery, Oulu University Hospital, Oulu; 6 Department of Surgery, Division of Orthopaedics and Traumatology, Satakunta Central Hospital, Pori and University of Turku, Turku, Finland

## Abstract

**Background and purpose:**

Robotic-assisted total knee arthroplasty (rTKA) has been proposed to improve component positioning but evidence on its effect on implant survival is limited. We aimed to compare implant survival between rTKA and conventional total knee arthroplasty (cTKA) overall and due to peri-prosthetic joint infection (PJI).

**Methods:**

We included all rTKAs performed in Finland from April 2021 until May 2023, all implanted with Triathlon TKA using MAKO robotic-arm assist. Propensity score matching was used to form a control group with similar characteristics for the same time period. Primary survival endpoint was first revision due to any cause within 12 months after the index surgery. PJI and non-PJI revisions were considered secondary outcomes. Kaplan–Meier (KM) analysis and Cox proportional hazards regression were used to assess implant survival and between-group contrasts hazard ratios (HR) with 95% confidence intervals (CIs), respectively.

**Results:**

1,318 fixed bearing rTKAs were compared with a control group of 1,318 cTKAs. Revision-free survival at 12 months after index surgery did not differ between groups. KM estimates were 99.1% (CI 98.5–99.7) for rTKA and 98.6% (CI 98.0–99.3) for cTKA with HR 0.61 (CI 0.28–1.34). The rTKA group demonstrated better PJI-free survival at 12 months (HR 0.29, CI 0.10–0.90). Overall, PJI was the most common reason for revision. At the end of available follow-up, the estimated overall survival was 97.7% (CI 94.9–100.0) for rTKA and 97.9% (CI 96.6–99.2) for cTKA.

**Conclusion:**

No difference in all-cause revision risk was observed between rTKA and cTKA groups. However, within the first 12 months, fewer revisions due to PJI occurred in the rTKA group.

Conventional total knee arthroplasty (cTKA) is an effective treatment for relieving pain in advanced knee osteoarthritis (OA) [1,2]. However, as many as 25% of patients are suffering from chronic pain and 18% are somewhat dissatisfied with their outcome [3]. Currently robotic-assisted TKA (rTKA) is increasingly utilized to improve the outcome of TKA. rTKA has been associated with better consistency of implant positioning and better alignment compared with cTKA, suggesting improved surgical precision. Knees operated on with robotic assistance had also statistically better patient-reported outcomes (PROM) than conventionally operated on knees although mostly not reaching minimal clinically important difference in PROMs used and rTKA has also been reported to reduce healthcare costs [4].

There is limited data on short-term revision rates after rTKA compared with cTKA. Introduction of usage of robotic assistance may have an impact on implant survival due to learning curve issues, and due to peri-prosthetic infections (PJI) due to extra machinery and personnel in the operating room, longer operating times, and additional skin incision due to extra pins. PJI is a devastating complication from patients’ point of view and the most common reason for revision [5], especially during the first postoperative year [6,7]. With the increased usage of rTKA, understanding its impact on revision and complication rates is crucial, and the cost-effectiveness before implementation as a routine in the daily clinic [8]. We aimed to assess early revision rates of rTKA and cTKA overall and due to PJI based on the Finnish Arthroplasty Register (FAR) and Finnish Care Register for Health Care (HILMO) data.

## Methods

### Study design

We designed a prospective observational cohort study with propensity score matched comparison of robot-assisted TKA and conventional TKA operation. A STROBE checklist for the cohort study is provided as supplementary material.

### Data source

The Finnish Care Register for Health Care (HILMO) is a national register for all Finnish healthcare and social services that gathers information on all healthcare services including diagnoses and surgical operations. Reporting TKAs to FAR and the Care Register is mandatory. Both registers are maintained by the Finnish Institute of Health and Welfare. Linking primary and revision operations and death of the patient is performed using a unique social security number. Since May 2014, implants have been identified by the electronic reading of reference codes perioperatively. Operative data is sent electronically to FAR and the Care Register. In May 2014, the data content of the FAR was examined and revised. The updated data now includes detailed information on items such as patient body mass index (BMI) and American Society of Anesthesiologists (ASA) class, surgical approach, and possible use of a navigation system or robotic-assisted surgery. All arthroplasty units deliver data; thus, the coverage of hospitals is 100%. According to the FAR, the completeness of data is > 98% for primary TKA and 92–95% of revision TKAs were reported to FAR in 2021 and 2022, and thus overall completeness of FAR has increased recently [5]. By linking Finnish Care Register data with FAR it is possible to include comorbidity data of the patients and count the Charlson index, and to increase completeness in identifying rTKAs. In Finland 4 hospitals (Kuopio University Hospital, Oulu University Hospital, Satakunta Central Hospital, and Mikkeli Central Hospital) use the MAKO robotic-arm-assisted Triathlon TKA (Stryker, Kalamazoo, MI, USA) in everyday practice. FAR and the Care Register collect data on TKA outcomes in Finland, including also whether the procedure was assisted with a robot arm or not.

### Data

**Figure 1:**
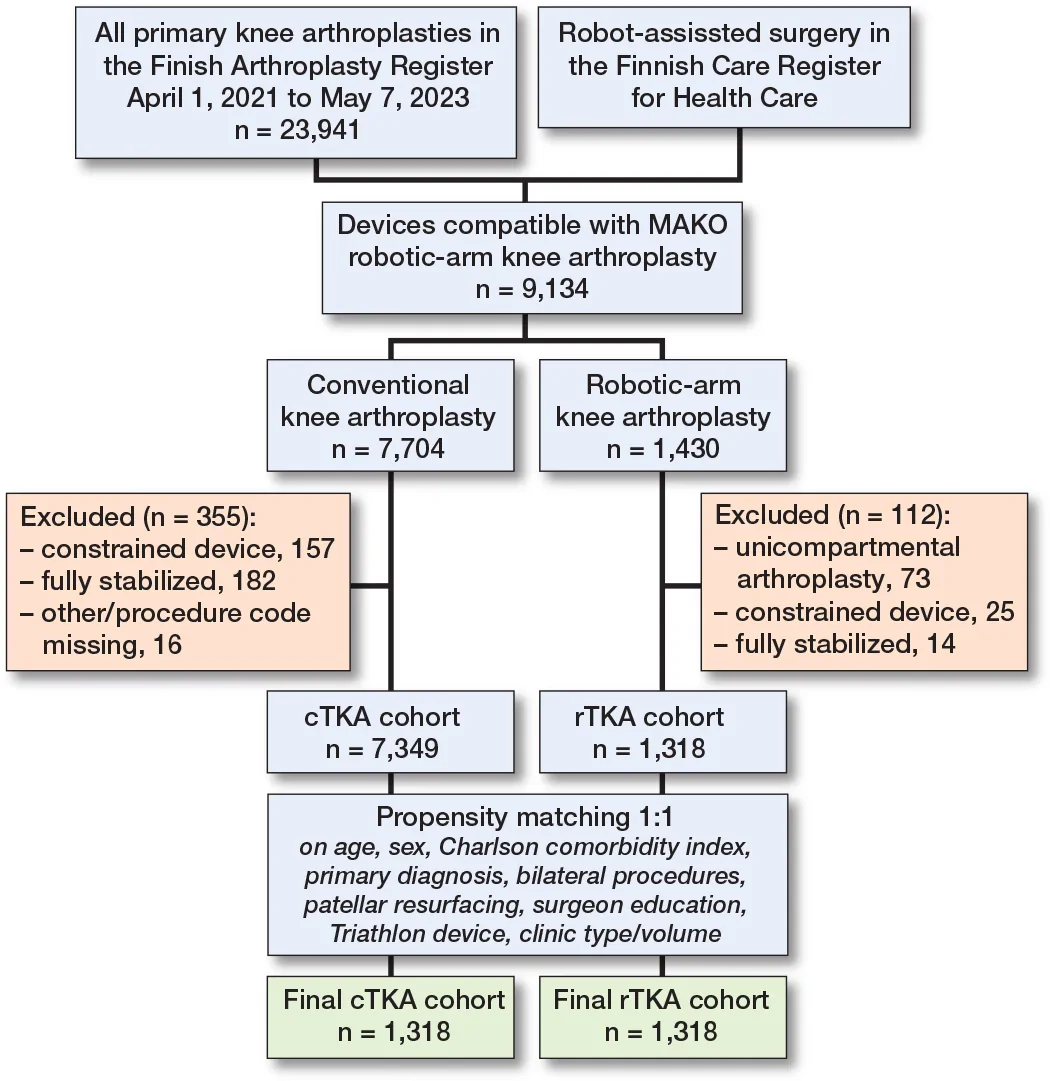
Patient flowchart.

Mako Total Knee SmartRobotics robotic-arm (MAKO) assisted TKA system (Stryker, Kalamazoo, MI, USA) utilizing a Triathlon device or MAKO unicompartmental knee arthroplasty (UKA) utilizing a Restoris MCK device are the only robotic-arm systems that are currently used in Finland. Primary MAKO rTKAs started in April 2021 and are currently used in 4 Finnish public hospitals: Satakunta Central Hospital, Mikkeli Central Hospital, Oulu University Hospital, and Kuopio University Hospital. From the FAR we extracted all primary TKAs with a MAKO-compatible device until May 2023 and identified robotic-arm-assisted procedures with FAR and the Care Register data (see Figure 1). All fixed-bearing rTKAs were included whereas UKAs and constrained models were excluded to form the rTKA cohort and, with identical criteria for inclusion and propensity-score matching, the cTKA cohort was formed. The matching was done on all available clinically important variables including age, sex, Charlson comorbidity index, primary diagnosis (primary OA or other), bilateral procedure (yes or no), patellar resurfacing (yes or no), surgeon education (orthopedic surgeon or resident/other), Triathlon device (cruciate retaining or posterior stabilized), and clinic type/volume (central/university/large-volume private hospitals or small-volume private clinic). Matching for these, surgeries for the final cohort were selected from a total of 21 different hospitals, including 3 university hospitals, 17 central/regional hospitals, and 1 large-volume public hospital. During the study period, the average annual volume of Triathlon cTKAs across included hospitals was 158 surgeries/year. For comparison, the average annual volume of Triathlon rTKAs was 157 surgeries/year in the 4 hospitals performing robotic-arm assisted surgeries during the same period.

### Study outcomes

The primary outcome was the first revision due to any cause occurring within the first year after index surgery in which one or more prosthesis components were removed, added, or exchanged for any reason. Revisions due to PJI were considered as a secondary outcome and revisions due to reasons other than PJI were considered as additional outcomes of interest. Further, we also explored implant survival for all-cause revision at the end of available follow-up. The median follow-up time was 11.5 months (range 0.1–25 months) in the rTKA cohort and 14.9 (range 0.1–25 months) in the cTKA cohort.

### Statistics

Our primary outcome was the short-term implant survival for all-cause revision between rTKA and cTKA cohorts within the first 12 postoperative months. Additionally, we compared implant survival for PJI and non-PJI causes between the 2 cohorts as secondary outcomes. As an exploratory analysis, we also report implant survival for all-cause revision at the end of available follow up for both cohorts.

Baseline characteristics of rTKA and cTKA cohorts were summarized using frequencies/group totals (percentages) for categorical variables and, due to skewed distributions, as medians with interquartile ranges (IQR) for continuous variables. To facilitate comparison of the baseline characteristics between cohorts, we computed standardized mean differences for all clinically relevant variables.

Overall implant survivorship probabilities with 95% confidence intervals (CI) were estimated using Kaplan–Meier (KM) analysis for both primary and key secondary outcomes. The patients were censored at data cutoff on May 7, 2023, or at the time of death. In the case of secondary outcomes, censoring was done also at the time of revisions due to causes other than those under observation. We treated bilateral operations as separate independent observations, because bilateral operation has previously been shown to have minimal effect on implant survival [9].

To estimate relative between-group hazards, Cox proportional hazards (PH) models were used to obtain hazard ratios (HR) with 95% CIs. The PH assumption for Cox analyses was assessed graphically from KM curves as well as by a test based on scaled Schoenfeld residuals [10,11]. Competing risk analysis was not performed because our primary analytical aim was to evaluate relative between-group hazards over a relatively short time period and mortality in both cohorts was essentially the same.

As a sensitivity analysis of our findings, we performed all comparisons between rTKA and cTKA cohorts separately for patients with primary OA as their main indication for surgery. This was done to ensure that the obtained estimates were not affected by inclusion of surgeries with diverse underlying reasons and would remain similar in a clinically more homogeneous population.

Statistical analyses were carried out using R statistical computing environment version 4.3.1 (R Core Team, 2016. R: A language and environment for statistical computing. R Foundation for Statistical Computing, Vienna, Austria. URL https://www.R-project.org/). R packages MatchIt, smd, survival, and survminer were used for propensity score matching, calculation of standardized mean differences, survival analysis, and visualization of KM curves, respectively. No variables included in the primary or secondary analyses contained missing values and thus no imputation was needed. Statistical significance of HRs was assessed primarily through 95% CIs whereas P values were treated as supplementary.

### Ethics, data sharing plan, funding, and disclosures

Ethical approval was granted (Dnro THL/2267/14.02.00/2022). This research received funding from the Päivikki and Sakari Sohlberg Foundation for statistical analyses. MSV reports funding from the Academy of Finland [grant number 322123]. The authors declare no conflict of interest regarding this research. Complete disclosure of interest forms according to ICMJE are available on the article page, doi: 10.2340/17453674.2026.46462

## Results

From the FAR we extracted all primary TKAs with a MAKO-compatible device until May 2023 (Figure 1). Among these, we identified all robotic-arm assisted procedures based on the procedural codes in the FAR and complemented the cohort with those reported to the Care Register (n = 9,134). Next, we excluded UKAs as well as all constrained prostheses (rTKA n = 112, cTKA n = 335) to arrive at our final cohort for rTKA (n = 1,318). After applying identical inclusion criteria for cTKA procedures (n = 7,349), using propensity score matching, we formed our cTKA cohort (n = 1,318) with characteristics similar to those of the rTKA cohort to be used as a reference group in the comparisons of implant survival. The median follow-up time was 11.5 months (range 0.1–25 months) in the rTKA cohort and 14.9 (range 0.1–25 months) in the cTKA cohort.

Median age of the patients was 68 (IQR 61–74) years in the rTKA group and 68 (IQR 61–74) years in the cTKA group; most were women in both groups (825/1,318 [63%] in rTKA and 815/1,318 [62%] in cTKA cohorts, respectively) and belonged to ASA class II (719/1,318 [55%] in rTKA vs 810/1,318 [62%] in cTKA cohorts). Median Charlson was 3 (IQR 2–5) points in therTKA and 3 (IQR 2–5) points in the cTKA group, whereas median BMI was 30 (IQR 26–34) and 30 (IQR 26–33), respectively. Primary OA was the leading indication (1,293/1,318 [98%] in rTKA and 1,300/1,318 [99%] in cTKA) and the use of patellar resurfacing was uncommon (77/1,318 [5.8%] with rTKA and 78/1,318 [5.9%] with cTKA) in both groups. Except for operation time, which was longer for rTKA (75 min, IQR 65–86) than for cTKA (61 min, IQR 51–73), no differences in baseline or surgical characteristics were observed between the 2 groups (Table 1).

**Table 1:** Demographics and clinical characteristics for robot-assisted TKA (rTKA) and matched non-robot-assisted TKA (cTKA) cohorts. Values are count (%) or as specified

Variable	rTKA (n = 1,318)	cTKA (n = 1,318)	SMD
Age, median (IQR)	68 (61–74)	68 (61–74)	0.03
Sex			
Male	493 (37)	503 (38)	0.02
Female	825 (63)	815 (62)	
ASA			0.14
I	81 (6.1)	77 (5.8)	
II	719 (55)	810 (62)	
III	510 (39)	426 (32)	
IV	8 (0.6)	5 (0.4)	
Charlson, median (IQR)	3 (2–5)	3 (2–5)	0.00
BMI, median (IQR)	30 (26–34)	30 (26–33)	0.00
Bilateral procedure	49 (3.7)	35 (2.7)	–0.06
Surgeon education			0.04
Orthopedic surgeon	1,314 (100)	1,315 (100)	
Resident or other	4 (0.3)	3 (0.2)	
Operating time [minutes], median (IQR)	75 (65–86)	61 (51–73)	–0.85
Diagnosis			0.04
Primary osteoarthrosis	1,293 (98)	1,300 (99)	
Other	25 (1.9)	18 (1.4)	
TKA with patellar resurfacing			0.00
No	1,241 (94)	1,240 (94)	
Yes	77 (5.8)	78 (5.9)	
Triathlon device			0.01
Cruciate retaining	1,301 (99)	1,300 (99)	
Posterior stabilized	17 (1.4)	18 (1.3)	

ASA = American Society of Anesthesiologists, BMI = body mass index, IQR = interquartile range, TKA = total knee arthroplasty, SMD = standardized mean difference.

### Outcome

**Figure 2:**
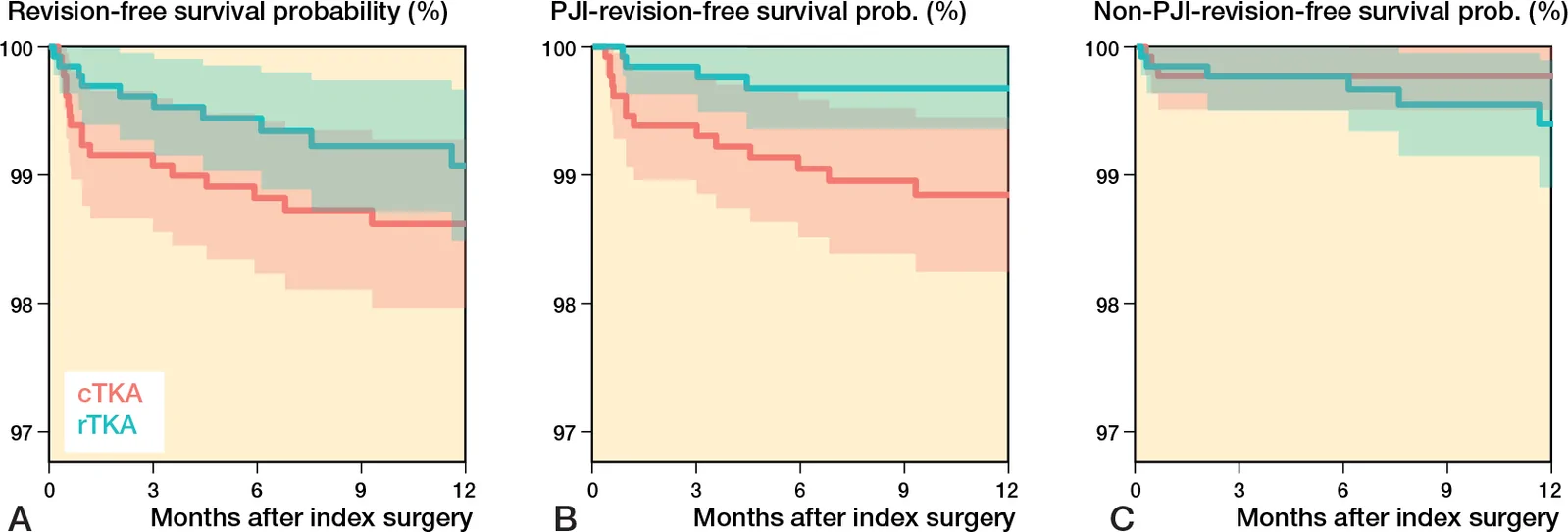
Kaplan–Meier curves for overall revision-free implant survival and revision due to periprosthetic joint infection (PJI) and revision due to causes other than PJI as an endpoint in robot-assisted total knee arthroplasty (rTKA) and non-robot-assisted total knee arthroplasty (cTKA) cohorts.

10 revisions were recorded in the rTKA group and 17 in the cTKA group within the first 12 months after the index surgery (Table 2). At this time point, the KM estimates for revision-free implant survival were 99.1% (CI 98.5–99.7) for the rTKA group and 98.6% (CI 98.0–99.3) for the cTKA group (HR 0.61, CI 0.28–1.34) (Figure 2A). While overall revision-free survival demonstrated no statistical difference between the groups, the greatest difference in implant survival was observed during the first postoperative month with rTKA, resulting in fewer revisions. At the end of the available follow-up (25 months), the estimated survival was 97.7% (CI 94.9–100.0) for the rTKA cohort and 97.9% (CI 96.6–99.2) for the cTKA cohort, (HR 0.66, CI 0.31–1.40).

**Table 2:** Hazard ratio (HR) estimates for primary (any cause revision) and secondary outcomes within 12 months after the index surgery

Outcome	rTKA n (%)	cTKA n (%)	HR (CI)
Primary outcome			
Any cause revision	10 (0.8)	17 (1.3)	0.61 (0.28–1.30)
Secondary outcomes			
PJI revision	4 (0.3)	14 (1.1)	0.29 (0.10–0.90)
Non-PJI revision	6 (0.5)	3 (0.2)	2.12 (0.53–8.50)

CI = 95% confidence interval; cTKA = non-robot-assisted (conventional) total knee arthroplasty; rTKA = robot-assisted total knee arthroplasty.

The rTKA cohort had better survival in terms of revision for PJI as endpoint with 4 recorded revisions compared with the cTKA cohort with 14 revisions due to PJI (HR 0.29, CI 0.10–0.90) (see Table 2). The estimated PJI-free survival at 12 months after the index surgery was 99.7% (CI 99.4–100.0) for the rTKA cohort and 98.8% (CI 98.2–99.5) for the cTKA cohort (Figure 2B). Similar to overall revision, the most notable difference in implant survival was observed during the first postoperative month. In the rTKA cohort, all revisions due to PJI took place within the first 6 postoperative months.

No difference in implant survival was observed between the rTKA (6 revisions) and cTKA (3 revisions) cohorts for revisions due to other causes than PJI (HR 2.1, CI 0.53–8.5) (see Table 2). The estimated revision-free survival within the first 12 postoperative months for causes other than PJI was 99.4% (CI 98.9–99.9) in the rTKA cohort and 99.8% (CI 99.5–100.0) in the cTKA cohort (Figure 2C).

The reasons for revision showed that non-PJI revisions, which accounted for 6/10 of all revisions in the rTKA group, included revisions due to periprosthetic fracture (1), malposition of component (1), tibiofemoral instability (1), pain (1), and other/missing causes (2) (Table 3). In the cTKA group, the non-PJI revisions (3/17) included only revisions due to periprosthetic fracture (1), and other/missing causes (2). There were 7 (0.5%) deaths in the rTKA group and 9 (0.7%) in the cTKA group within the first year, so mortality was similar in both groups.

**Table 3:** Adverse outcomes within the first 12 months from the index surgery. Values are count (%)

Outcome	rTKA (n = 1,318)	cTKA (n = 1,318)
Revision	10 (0.8)	17 (1.3)
Infection	4 (0.3)	14 (1.1)
Periprosthetic fracture	1 (0.1)	1 (0.1)
Malposition of component	1 (0.1)	0 (0.0)
Tibiofemoral instability	1 (0.1)	0 (0.0)
Pain	1 (0.1)	0 (0.0)
Other / reason missing	2 (0.2)	2 (0.2)
Death	7 (0.5)	9 (0.7)

For abbreviations, see Table 2.

All aforementioned findings remained similar in our sensitivity analysis consisting only of patients with primary OA as their main indication for surgery (Supplementary data).

## Discussion

We aimed to compare implant survival between rTKA and cTKA and showed no difference in overall survival,but that rTKA cohort had better survival in terms of revision for PJI.

Compared with our study, Ofa et al. found a higher revision rate for the cTKA group than for the rTKA (odds ratio [OR] 1.21) [12]. Aggarwal et al. found a reduced risk for PJI (OR 0.027), periprosthetic dislocation (OR 0.117), periprosthetic fracture (OR 0.28), and periprosthetic mechanical complications (OR 0.315) in rTKA in comparison with cTKA [13]. In a national cohort there was no difference between rTKAs and cTKA regarding PJI, prosthesis breakage, dislocation, loosening, and periprosthetic fracture, or overall revision risk [14]. In the Australian Orthopedic Association National Joint Replacement Registry there were no differences in revision rates between cTKAs and rTKAs [15]. Previously, in 3 smaller patient cohorts, 1 smaller RCT, and 1 meta-analysis comparing cTKA and MAKO rTKA, there were no differences concerning complications but less postoperative pain, improved early mobilization, surgical precision, and better alignment was reported [16–20].

In theory, the rTKA method might aid in reducing septic complications due to less hand contact with the wound intraoperatively, and there may also be less surgical soft tissue trauma, fewer releases, and less bone exposure [21]. Further, opening of the intramedullary canal is not needed in rTKA. However, these effects could be negated by the extra personnel needed to operate the robot in the operating room, slightly longer operation time on average, and possible extra skin wounds if intraoperative pins are inserted outside the parapatellar incision. In our study, somewhat different non-PJI revision indications in the rTKA compared with the cTKA group may be associated with the learning curve of first rTKAs. Unfortunately, we have no further information on the cases and the number of revisions is too low to draw meaningful conclusions.

Overall, there have been a few studies comparing rTKA and cTKA using 1 specific robotic system and even fewer studies with the MAKO system. It is important to assess each rTKA system separately as these may differ substantially [19]. Many studies on MAKO rTKAs focus on functional outcomes and surgical accuracy, and not on early to mid-term revision rates [20].

### Strengths

The strength of our study was that we were able to assess a single robotic-assisted system. Further, our study was based on large national arthroplasty register data which includes all robotic-assisted TKAs in Finland up to the end of the study period. This enables the assessment of how the modern method of knee arthroplasty has affected the short-term complication rates and how well the new technology has fared in Finland. In the absence of large randomized controlled trials, unselected registers are the best source of data concerning surgical complications of rTKA.

### Limitations

Our data represents the first national results of a new technology in Finland, and therefore certainly includes surgeons with a learning curve. Also, during the learning phase easier knees with less malalignment or contracture might have been selected, although this may not always be the case. Further, during the learning curve, rTKA operation time may also be longer compared with cTKA [21]. On the other hand, only specialists are allowed to perform MAKO rTKA after proper education, and only public large-volume units perform rTKAs in Finland to date. While surgeon seniority and hospital size were not direct risk factors for PJI in a previous register study from FAR data [22] the pronounced effect in this case could present as a confounding factor to revision risk and contradictory findings from register studies have been reported [23].

The overall number of rTKA revisions at this point was very small, only 10 revisions. Also, the follow-up time of 12 months was not reached in all of the rTKAs and cTKAs and as the rTKA group is not vast this could affect the results of full 12-month revision rates. Further, residual confounding may in part explain the decreased PJI risk of the rTKA group. We also lack information regarding alignment techniques used and their effect on revision risk. We did not have any data on radiographic outcomes, on patient-reported outcome measures (PROMs), or on the cost-effectiveness of rTKA. Register completeness of revision TKAs is still lacking and some cases may be missing from analysis, especially those performed on call, such as revisions for PJIs.

### Conclusion

Primarily our study demonstrated no difference in overall survival between robotic-arm-assisted TKAs and conventional TKAs. A secondary result was that the rTKA cohort had better survival in terms of revision for PJI, but no difference was found in other reasons for revision.

*In perspective,* there is a need for longer follow-up time and larger cohorts are needed to assess any superiority of either technique.

### Supplementary data

Sensitivity analyses (Tables S1–S3 and Figure S1) are available as Supplementary data on the article home page, doi: 10.2340/17453674.2026.46462

## Supplementary Material



## Notes

KA and ES wrote the manuscript draft, MV performed statistical analyses, KM designed the study, and all authors contributed to the final the manuscript.

The authors would like to thank Mr Jaason Haapakoski and MD Jutta Järvelin from the Finnish Institute for Health and Welfare for their invaluable help with the data.

Handling co-editors: Marianne Westberg and Robin Christensen

*Acta* thanks Ove Nord Furnes and Anders Troelsen for help with peer review of this manuscript.
