# Supplementary material for: Early revision rate in robotic-assisted versus conventional total knee arthroplasty: a prospective cohort study 2021–2023 from the Finnish Arthroplasty Register
Source: Acta Orthop. 2026 Sep 3;97:609–14. doi: 10.2340/17453674.2026.46462 (PMC13543273; doi:10.2340/17453674.2026.46462)
Supplement: Supplementary file 1 [file ActaO-97-46462-s1.pdf]

Supplementary data

Sensitivity analysis

Tables

Supplementary Table S1. Demographics and clinical characteristics of patients with primary osteoarthritis as the main indication for surgery in robot-assisted total knee arthroplasty (rTKA) and non-robot-assisted total knee arthroplasty (cTKA) cohorts.

Variable	rTKA (n=1,293)	cTKA (n=1,300)	SMD
Age, median (IQR)	68 (61–74)	68 (61–74)	0.03
Sex, n (%)			
Male	480 (37.1)	491 (37.8)	0.01
Female	813 (62.9)	809 (62.2)	
ASA, n (%)			0.15
I	78 (6.1)	76 (5.8)	
II	705 (54.5)	803 (61.8)	
III	502 (38.8)	416 (32.0)	
IV	8 (0.6)	5 (0.4)	
Charlson, median (IQR)	3 (2–5)	3 (2–5)	0.00
BMI, median (IQR)	30.4 (26.9–34.3)	30.3 (26.6–33.8)	-0.01
Bilateral procedure, n (%)	48 (3.7)	35 (2.7)	-0.06
Surgeon education			0.04
Orthopaedic surgeon	1,289 (99.7)	1,297 (99.8)	
Resident or other	4 (0.3)	3 (0.2)	
Operating time [min], median (IQR)	75 (65–86)	61 (51–73)	-0.85
Operation TKA, n (%)			0.02
without patellar resurfacing	1,222 (94.5)	1,224 (94.2)	
with patellar resurfacing	71 (5.5)	76 (5.8)	
Triathlon device, n (%)			0.01
Cruciate retaining (CR)	1,277 (98.8)	1,283 (98.7)	
Posterior stabilized (PS)	16 (1.2)	17 (1.3)	

ASA = American Society of Anesthesiologists, BMI = body mass index, IQR = Interquartile range, TKA = total knee arthroplasty, SMD = standardized mean difference

Supplementary Table S2. Hazard ratio (HR) estimates for primary (any cause revision) and secondary outcomes among patients with primary osteoarthritis as their primary indication for surgery. CI denotes confidence interval.

	rTKA (n=1,293)		cTKA (n=1,300)			
Outcome	No. of patients	(%)	No. of patients	(%)	HR	(95% CI)
Primary outcome						
All-cause revision	10	(0.8)	17	(1.3)	0.62	(0.28–1.34)
Secondary outcomes						
PJI revision	4	(0.3)	14	(1.1)	0.30	(0.10–0.90)
Non-PJI revision	6	(0.5)	3	(0.2)	2.13	(0.53–8.53)

Supplementary Table S3. Adverse outcomes within the first 12 months from the index surgery among patients with primary osteoarthritis as their primary indication for surgery.

Outcome, n (%)	rTKA (n=1,293)	cTKA (n=1,300)
Revision	10 (0.77)	17 (1.31)
Infection	4 (0.31)	14 (1.08)
Periprosthetic fracture	1 (0.08)	1 (0.08)
Malposition of component	1 (0.08)	0 (0.00)
Tibiofemoral instability	1 (0.08)	0 (0.00)
Pain	1 (0.08)	0 (0.00)
Other / reason missing	2 (0.15)	2 (0.15)
Death	7 (0.54)	9 (0.69)

Figures

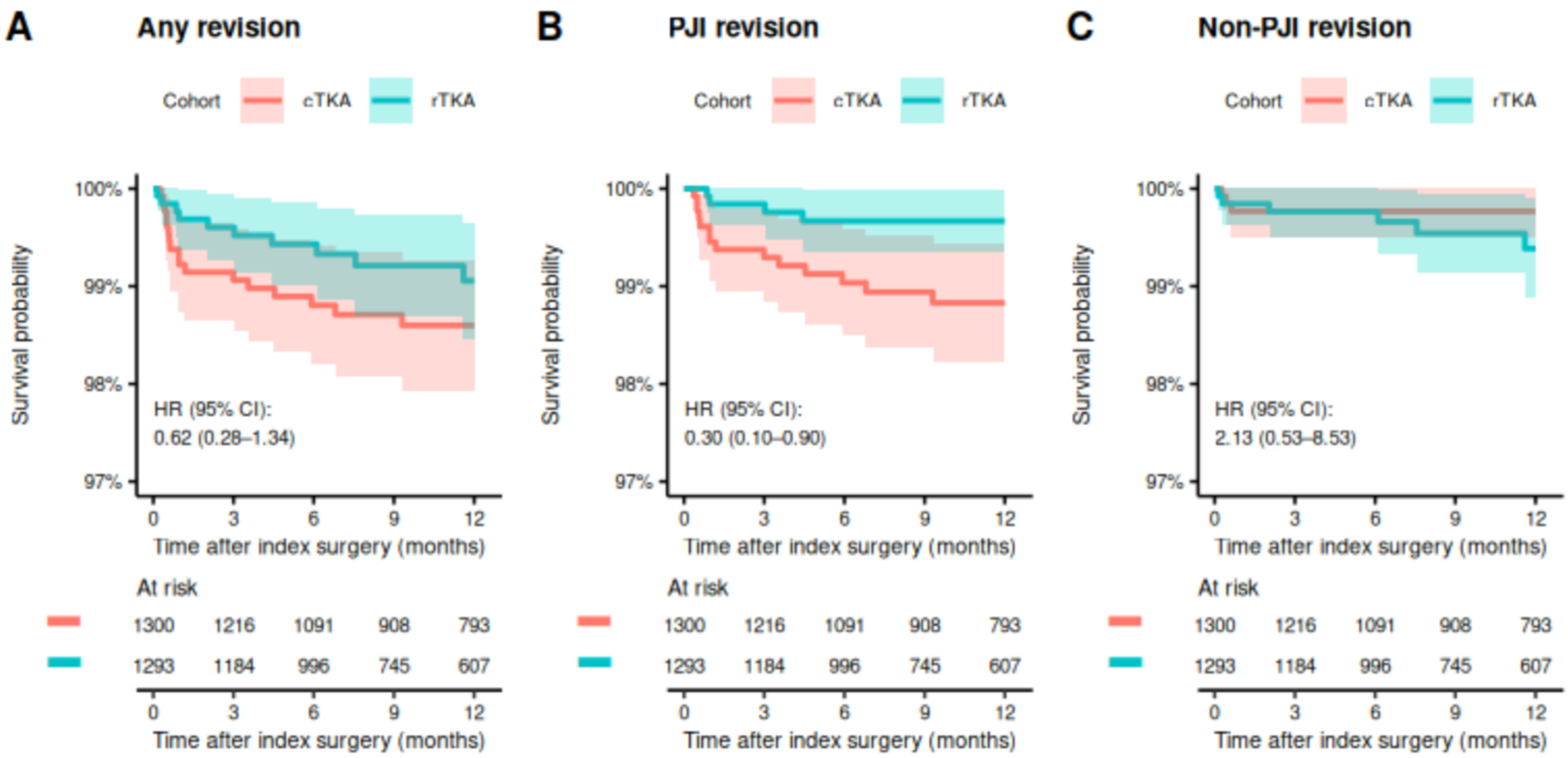

Supplementary Figure S1. Kaplan-Meier curves for A) overall revision-free implant survival and B) revision due to periprosthetic joint infection (PJI) or C) revision due to causes other than PJI as an endpoint among patients with primary osteoarthritis as their main indication for surgery in robot-assisted total knee arthroplasty (rTKA) and non-robot-assisted total knee arthroplasty (cTKA) cohorts.
